# On the Treatment of Pneumonia

**Published:** 1919

**Authors:** 


***On the Treatment of Pneumonia.*** By ELLIOT DICKSON. *The British Medical Journal*, October 19, 1918.

Dr. Elliot Dickson, in speaking of the slight progress made in the treatment of pneumonia, says that enough stress cannot be laid on the " enormous importance of rest ". Cardiac weakness is so pronounced that the patient can actually die from the effort of sitting up in bed.

Following the doctrine of Professor Greenfield, Dr. Dickson considers that there are two essential parts in the treatment.

(1) Absolute rest — the value of which cannot be over-estimated.

(2) All cases are given tincture of strophantus, from the time the diagnosis is made, in sufficient doses to keep the pulse satisfactory. Early dosage is extremely important; it is absolutely wrong to wait for signs of heart failure.

The dose of strophantus varies with individual reaction to the pneumotoxin.

Strychnin the author considers as positively harmful. Heroin, 1/6 grain hypodermically, is beneficial. It quiets the cough and induces sleep.

It should be remembered that the active principle of strophantin readily undergoes decomposition when diluted in water, so it should be prescribed as the tincture alone, and not made up with water in bulk.

